# Control of epitaxial relationships of ZnO/SrTiO_3_ heterointerfaces by etching the substrate surface

**DOI:** 10.1186/1556-276X-8-23

**Published:** 2013-01-10

**Authors:** Caihong Jia, Yonghai Chen, Xianglin Liu, Shaoyan Yang, Weifeng Zhang, Zhanguo Wang

**Affiliations:** 1 Key Laboratory of Semiconductor Material Science, Institute of Semiconductors, Chinese Academy of Science, P.O. Box 912, Beijing, 100083, People’s Republic of China; 2 Key Laboratory of Photovoltaic Materials of Henan Province and School of Physics and Electronics, Henan University, Kaifeng, 475004, People’s Republic of China

**Keywords:** ZnO, SrTiO_3_, Epitaxial

## Abstract

Wurtzite ZnO thin films with different epitaxial relationships are obtained on as-received and etched (001), (011), and (111) SrTiO_3_ (STO) by metal-organic chemical vapor deposition (MOCVD). ZnO films exhibit nonpolar (1120) orientation with in-plane orientation relationship of <0001>_ZnO_//<110>_STO_ on as-received (001) STO, and polar *c*-axis growth with <1100>_ZnO_//<110>_STO_ on etched (001) STO substrates. ZnO films change from polar (0001) to semipolar (1012) oriented on as-received and etched (011) STO. On as-received and etched (111) STO, ZnO films show the same growing direction of polar (0001), but different in-plane orientations with 30° rotation. The change of epitaxial relationship of ZnO films on as-received and etched (001), (011), and (111) STO substrates is accompanied with the increase of lattice mismatch, decrease of bond density, and increase of substrate surface roughness. In other words, the epitaxial relationships of ZnO/STO heterointerfaces can be controlled by etching the substrates. These results show that polar, nonpolar, and semipolar ZnO films for different applications can be grown epitaxially on STO substrates by MOCVD.

## Background

Growth direction is a key element to determine the electrical and optical properties of ZnO thin films, and different orientations are demanded for various applications [1,2]. Polar ZnO films with a *c*-axis perpendicular to the growth plane are required for the high electron mobility transistor structure, which depends on the realization of a high-density two-dimensional electron gas using electric polarization effects. The nonpolar and semipolar ZnO films with a horizontal and inclined *c*-axis are expected to show higher emission efficiency in light-emitting diodes by eliminating or reducing the spontaneous and piezoelectric polarization fields [3-5].

SrTiO_3_ (STO) single crystal substrates have been widely used to deposit functional oxide films with superconductivity, ferroelectricity, and ferromagnetism owing to lattice match. Compared with other common substrates for ZnO growth, the integration of wurtzite ZnO and perovskite STO combines the rich properties of perovskites together with the superior optical and electrical properties of wurtzites [6-9]. Thus, the ZnO/STO heterojunction is expected to be applied in new multifunctional devices due to carrier limitation and coupling effect. On the other hand, it is found that the pretreatment method of (001) STO single crystal substrates will significantly influence the growth behaviors of thin films. For example, Pb(Zr,Ti)O_3_[10] and (Sr,Ba)Nb_2_O_6_[11] films show different growth modes and orientations on the TiO_2_- and SrO-terminated surfaces of (001) STO substrates, whereas SrRuO_3_[12] and BaTiO_3_[13] films exhibit different initial morphology and crystallinity on the as-received and etched (001) STO substrates, respectively. However, there is little research about the growth behavior of ZnO films on as-received and etched (001), (011), and (111) STO substrates. Furthermore, the control of epitaxial relationships for ZnO on STO has not been investigated in detail.

In this paper, polar, nonpolar, and semipolar ZnO films are obtained on as-received and etched (001), (011), and (111) STO substrates by metal-organic chemical vapor deposition (MOCVD). X-ray *θ*-2*θ* and Ф scannings are performed to determine the out-of-plane and in-plane epitaxial relationships between ZnO films and STO substrates.

## Methods

The substrates used were (001), (011), and (111) STO single crystal wafers with sizes of 10 × 5 × 0.5 mm^3^. The as-received STO substrates were polished and cleaned by an organic solution, while the etched substrates were further conducted in buffered HF solutions at room temperature. ZnO films were grown on both as-received and etched STO substrates by a home-designed and made vertical low-pressure MOCVD reactor. Bubbled diethylzinc (DEZn) and pure oxygen were the reactants, and nitrogen gas was used as the carrier gas. The samples were grown at 600°C for 30 min with the same bubbled diethylzinc flux and carrier gas flux of oxygen. The flow rate of the pure oxygen gas was set at 1 slpm, and the flow rate of DEZn was set at 16 sccm. The pressure of the chamber was kept at 76 Torr. The epitaxial relationships were determined by X-ray *θ*-2*θ* (X’Pert Pro MPD, PANalytical, Almelo, The Netherlands) and Ф scannings (TTR III, Rigaku, Tokyo, Japan) with CuKα radiation.

## Results and discussion

**Figure 1:**
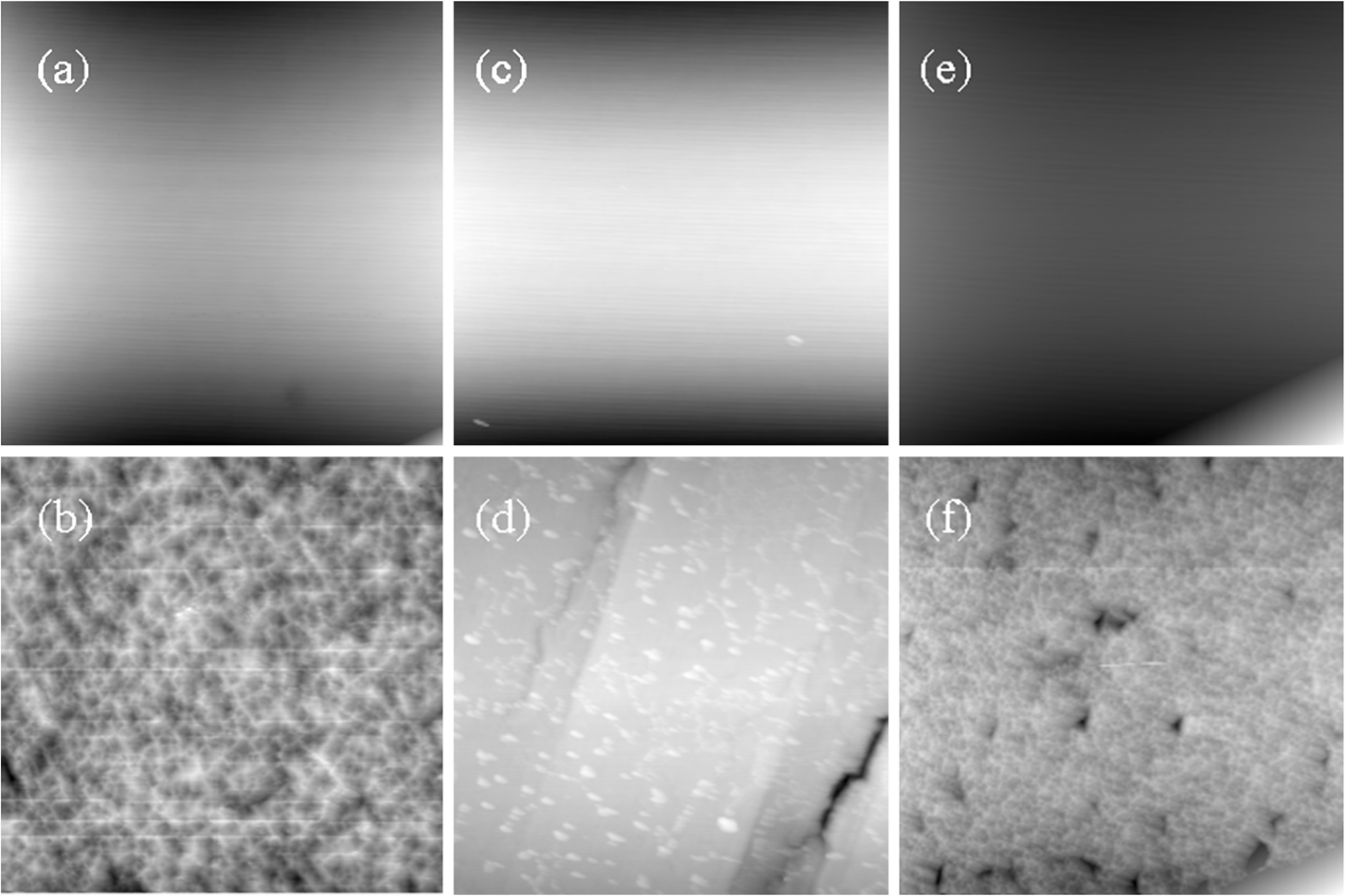
**AFM images (10 × 10 μm**^**2**^**).**The as-received (**a**, **c**, **e**) and etched (**b**, **d**, **f**) (001) (**a**, **b**), (011) (**c**, **d**), and (111) (**e**, **f**) STO substrates.

Figure 1 shows the surface images of as-received and etched STO substrates taken by an atomic force microscope (AFM). It can be clearly seen that the STO surface varies from smooth for as-received to rough for etched. The surface roughness of as-received STO substrates is about 1 nm, while the etched STO surface is full of pits or trenches with a surface roughness of around 20 nm. Although some reports show that the surface of HF-etched STO is atomically flat with Ti-terminated surface since Sr atom is much more sensitive to HF attack than Ti atom [14], the etched STO surface in the present case is full of pits or trenches. The STO used in this work may not be a perfect single crystal and is assumed to be made up of nanograins [15]. The HF solution permeates into the grain boundaries and dissolves Sr atoms on the lateral sides. As etching proceeds, the grains shrink and the grain boundaries widen in size, leading to the appearance of pits or trenches. The tilted angles of pits or trenches from the surface are estimated from AFM to be 56.4°, 41.8°, and 64.0° on etched (001), (011), and (111) STO substrates, respectively. The pits and/or trenches may serve as patterned substrates to control the growth direction of ZnO films, which is essentially important for practical applications.

X-ray *θ*-2*θ* and Ф scans were performed to identify the out-of-plane and in-plane orientation relationships between the films and substrates. In a Ф scan, the number of peaks corresponds to the number of planes for a particular family that possesses the same angle χ (0°< χ < 90°) with the crystal surface, while the separation between peaks correlates with the angular separation between the corresponding projections of the normals to the scanning family onto the crystal surface. The Ф angles of the ZnO films are respectively corrected by the Ф scan of the STO substrates.

**Figure 2:**
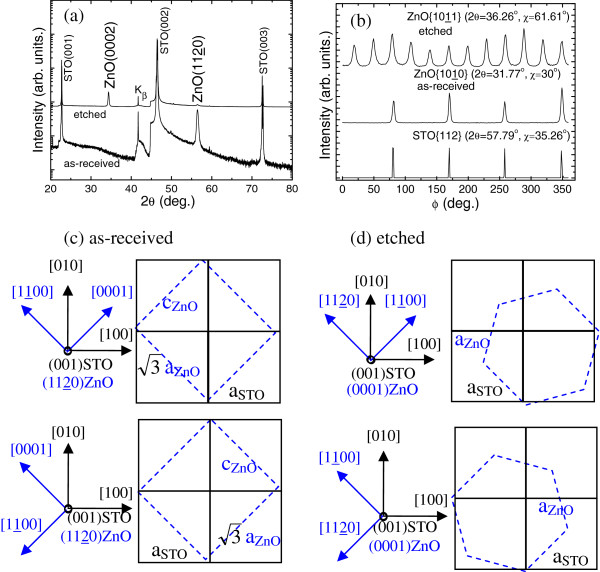
**ZnO films on as-received and etched (001) STO substrates.** X-ray *θ*-2*θ* (**a**) and Ф (**b**) scanning patterns and atomic arrangements (**c**, **d**).

It can be seen from Figure 2a that ZnO films show nonpolar (1120) and polar (0001) orientations on as-received and etched (001) STO substrates, respectively. We first discuss the epitaxial relationship of (1120) ZnO on as-received (001) STO. Several groups have obtained (1120) ZnO epitaxial films on (001) STO, but suppose one-, two-, or four-domain epitaxy [7-9,16]. In order to clarify the epitaxial relationship of (1120)ZnO/(001) STO in the present work, we performed the Ф scans of ZnO {1010} and STO {112} families, as shown in Figure 2b. In single crystal (1120) ZnO, only two crystal planes in the ZnO {1010} family have the same angle with the surface (χ = 30°), and two peaks separated by 180° are expected in ZnO {1010} Ф patterns, which is just the case in single-domain (1120) ZnO on *r*-sapphire [17]. However, the reflections from the ZnO {1010} family show four peaks separated by 90°, implying that two domains perpendicular to each other coexist in the film plane. Furthermore, the peak positions in the Ф scans of ZnO {1010} (2*θ* = 31.77°, χ = 30°) and STO {112} (2θ = 57.79°, χ = 35.26°) coincide, implying that their zone axes are parallel to each other, that is, <0001>_ZnO_∥<110>_STO_, as shown in Figure 2c. In addition, the lattice mismatches are −5.7% ( $\frac{c_{\text{ZnO}}-\sqrt{2}a_{\text{STO}}}{\sqrt{2}a_{\text{STO}}}$), 1.9% ( $\frac{\sqrt{3}a_{\text{ZnO}}-\sqrt{2}a_{\text{STO}}}{\sqrt{2}a_{\text{STO}}}$) and −1.8% ( $\frac{\sqrt{{\left(\sqrt{3}a_{\text{ZnO}}\right)}^{2}+c_{\text{ZnO}}^{2}}-2a_{\text{STO}}}{2a_{\text{STO}}}$) along the directions of <0001>_ZnO_, <1100>_ZnO_, and <1101>_ZnO_ in the film plane, respectively.

Similarly, the in-plane orientation relationships for (0001) ZnO films on etched (001) STO can also be achieved from X-ray Ф scanning. Figure 2b displays 12 peaks separated by 30° for the ZnO {1011} family, which has six planes intersecting the surface at 61.6°. It indicates that two domains with 30° rotation coexist. Comparing the peak positions of the ZnO {1011} (2*θ* = 36.26°, χ = 61.61°) and STO {112} (2*θ* = 57.79°, χ = 35.26°), the in-plane orientation relationship is demonstrated to be <1120>_ZnO_//<110>_STO_ for (0001) ZnO on etched (001) STO substrates, and the atomic arrangements are shown in Figure 2d. The lattice mismatch in the direction of <1100>_ZnO_ is 1.9% ( $\frac{\sqrt{3}a_{\text{ZnO}}-\sqrt{2}a_{\text{STO}}}{\sqrt{2}a_{\text{STO}}}$), whereas in the direction of <1120>_ZnO_, a higher order matching with a mismatch of −1.9% can also be found for seven ZnO over six STO unit cells. The higher order matching has been proposed for the epitaxial growth in large lattice mismatch system [18], but the lower order matching is regarded as the leading growth mechanism. Although the lattice mismatch of the (1120) and (0001) ZnO with (001) STO are almost the same along <1100>_ZnO_, (0001)-oriented films are obtained on etched (001) STO. This result is considered to be related to the fact that ZnO films tend to be oriented in the (0001) direction even on amorphous substrates [19], implying that the restriction of substrates decreases and the surface energy becomes dominant for the growth of ZnO films on etched (001) STO. As a result, the (0001) plane having the lowest surface energy, the close-packing plane tends to be oriented on etched (001) STO substrates.

**Figure 3:**
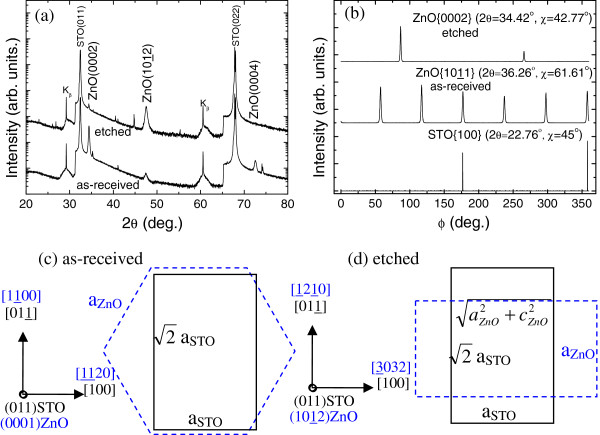
**ZnO films on as-received and etched (011) STO substrates.** X-ray *θ*-2*θ* (**a**) and Ф (**b**) scanning patterns and atomic arrangements (**c**, **d**).

Figure 3a shows that ZnO films exhibit (0002) and (1012) preferred orientations on as-received and etched (011) STO substrates. The angle between (1012) and (0002) is calculated to be 42.77°, which corresponds to the tilted angle of the trench in etched (011) STO (41.8°, as shown in Figure 1d). This phenomenon is similar to that of GaN on patterned (001) Si substrates [20]. The ZnO films on as-received (011) STO show similar X-ray *θ*-2*θ* and Ф scanning patterns with other reports [6,7], and the atomic arrangements are shown in Figure 3c. The in-plane orientation relationship obtained was <1100>_ZnO_∥<011>_STO_ by comparing the Ф scanning peak positions of ZnO {1011} (2*θ* = 36.26°, χ = 61.61°) and STO {100} (2*θ* = 22.76°, χ = 45°). The lattice mismatches are 1.9% ( $\frac{\sqrt{3}a_{\text{ZnO}}-\sqrt{2}a_{\text{STO}}}{\sqrt{2}a_{\text{STO}}}$) and −16.8% ( $\frac{a_{\text{ZnO}}-a_{\text{STO}}}{a_{\text{STO}}}$) along the directions of <1100>_ZnO_ and <1120>_ZnO_ in the film plane, respectively. For (1012) ZnO films on etched (011) STO, the in-plane orientation relationship obtained was<1210>_ZnO_∥<011>_STO_ by comparing the Ф scanning peak positions of ZnO {0002} (2*θ*= 34.42°, χ = 42.77°) and STO {100} (2*θ* = 22.76°, χ = 45°). The lattice mismatches are −41.2% ( $\frac{a_{\text{ZnO}}-\sqrt{2}a_{\text{STO}}}{\sqrt{2}a_{\text{STO}}}$) and 57.1% ( $\frac{\sqrt{a_{\text{ZnO}}^{2}+c_{\text{ZnO}}^{2}}-a_{\text{STO}}}{a_{\text{STO}}}$) along the directions of <1120>_ZnO_ and <3032>_ZnO_ in the film plane, respectively. Compared with ZnO films on the as-received (011) STO, much larger lattice mismatches are found for those on etched (011) STO substrates.

**Figure 4:**
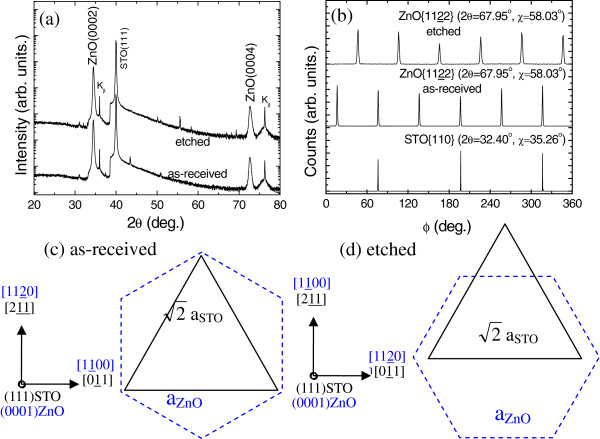
**ZnO films on as-received and etched (111) STO substrates.** X-ray *θ*-2*θ* (**a**) and Ф (**b**) scanning patterns and atomic arrangements (**c**, **d**).

Figure 4a shows that ZnO films exhibit a *c*-axis perpendicular to the growth plane on both as-received and etched (111) STO substrates. Only six peaks are observed for the ZnO {1122} family, which has six crystal planes with the same angle as the growth plane (χ = 58.03°), as shown in Figure 4b. Thus, both ZnO films are single-domain epitaxy on as-received and etched (111) STO, which exhibit a 30° rotation of the in-plane orientation. From the relative position of ZnO {1122} (2*θ* = 67.95°, χ = 58.03°) and STO {110} (2*θ* = 32.40°, χ = 35.26°) families, the in-plane relationships obtained was <1100>_ZnO_∥<011>_STO_ and <1120>_ZnO_∥<011>_STO_ on as-received and etched (111) STO substrates, respectively. The atomic arrangements in the heterointerface of (0002)ZnO/(111)STO are shown in Figure 4c, d. The lattice mismatch is 1.91% ( $\frac{\sqrt{3}a_{\text{ZnO}}-\sqrt{2}a_{\text{STO}}}{\sqrt{2}a_{\text{STO}}}$) along the direction of <1100>_ZnO_ on as-received (111) STO, while the lattice mismatch is about 17.7% ( $\frac{2a_{\text{ZnO}}-\sqrt{2}a_{\text{STO}}}{\sqrt{2}a_{\text{STO}}}$) along the direction of <1120>_ZnO_ on etched (111) STO. Surprisingly, the lattice mismatch increases a lot, but high quality with single-domain epitaxy is still preserved on etched (111) STO substrates. A similar phenomenon is also found in (0001) ZnO films on (111) BaTiO_3_ pesudo-substrates [21]. The interface of ZnO on etched (111) STO is supposed to be incoherent, and the interface chemical energy plays a more important role than interface elastic energy for a large lattice mismatch system; thus, the excessive interface stress induces the rotation of ZnO domains.

**Figure 5:**
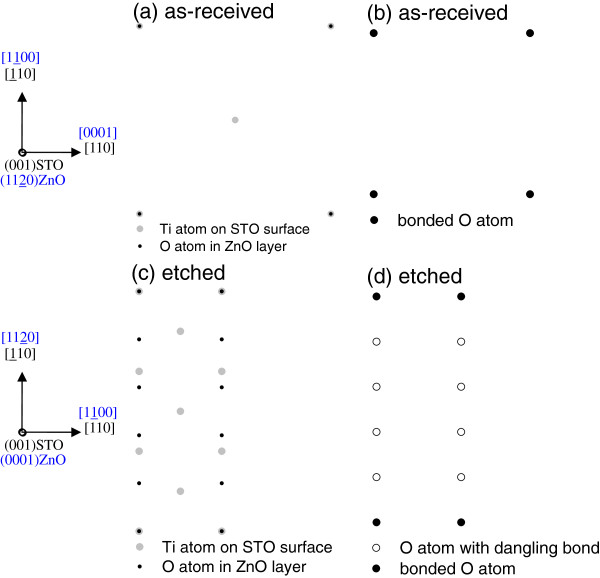
**The ZnO/(001)STO interface.** Schematic top views (**a**, **c**) and distribution of O atoms bonded to Ti atoms (**b**, **d**) of the ZnO/(001)STO interface, in which (**a**, **b**) are on as-received STO while (**c**, **d**) are on etched STO. Only the O atoms and Ti atoms closest to the interface are shown in (**a**, **c**).

**Figure 6:**
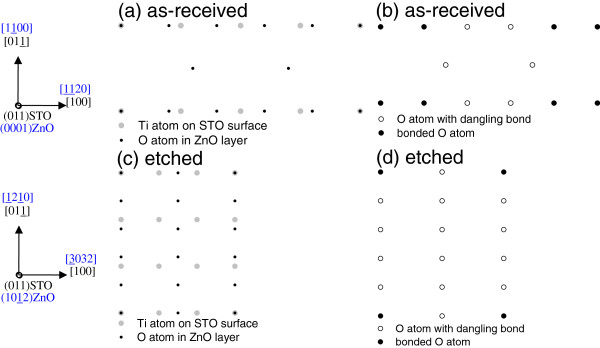
**The ZnO/(011)STO interface.** Schematic top views (**a**, **c**) and distribution of O atoms bonded to Ti atoms (**b**, **d**) of the ZnO/(011)STO interface, in which (**a**, **b**) are on as-received STO while (**c**, **d**) are on etched STO. Only the O atoms and Ti atoms closest to the interface are shown in (**a**, **c**).

**Figure 7:**
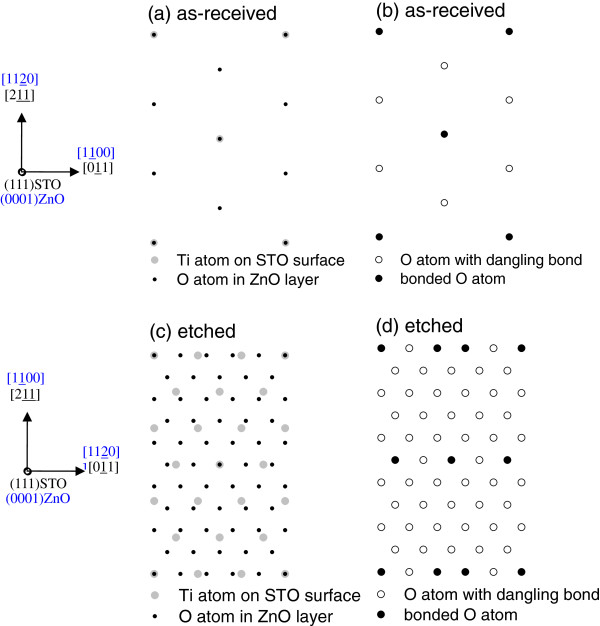
**The ZnO/(111)STO interface.** Schematic top views (**a**, **c**) and distribution of O atoms bonded to Ti atoms (**b**, **d**) of the ZnO/(111)STO interface, in which (**a**, **b**) are on as-received STO while (**c**, **d**) are on etched STO. Only the O atoms and Ti atoms closest to the interface are shown in (**a**, **c**).

Interestingly, all ZnO films prefer to grow with a much larger lattice mismatch on etched (001), (011), and (111) STO substrates. It is supposed that the interface dominates the film growth on as-received and etched STO, so it is essential to estimate the interface bond densities for each ZnO/STO heterointerface. To estimate the interface bond densities for each in-plane epitaxial relationship [22], we consider the in-plane atomic arrangements at the ZnO/STO interface for the case of as-received and etched STO surfaces. Figures 5a, c, 6a, c, and 7a, c show schematic top views of the ZnO/STO interfaces on as-received and etched (001), (011), and (111) STO substrates, respectively. In these figures, only the O atoms and Ti atoms closest to the interface are shown. Due to the large in-plane lattice mismatch between ZnO and STO, the arrangements of Ti-O bonds show the superstructure. In Figures 5b, d, 6b, d, and 7b, d, Ti-O bonds and dangling bonds are indicated by closed and open circles, respectively. Accordingly, the bond densities obtained were 3.41 × 10^14^ and 1.09 × 10^14^ cm^−2^ on as-received and etched (001) STO substrates, 3.28 × 10^14^ and 0.50 × 10^14^ cm^−2^ on as-received and etched (011) STO substrates, and 3.65 × 10^14^ and 1.31 × 10^14^ cm^−2^ on as-received and etched (111) STO substrates, respectively. Obviously, comparing with those on as-received STO, the bond density decreases greatly for ZnO films on etched STO. It is consistent with the fact that the substrate surface changes from smooth for as-received STO to rough for etched STO, as shown in Figure 1. With increasing substrate surface roughness, it becomes difficult to bond ZnO films and etched STO substrates, and the bond density decreases while the lattice mismatch increases largely for ZnO on etched STO. Therefore, the epitaxial relationship of ZnO/STO heterointerfaces can be controlled by etching the substrates.

## Conclusions

In summary, epitaxial ZnO thin films have been obtained on as-received and etched (001), (011), and (111) STO substrates by MOCVD, and the epitaxial relationships were determined. It is interesting that ZnO films exhibit nonpolar (1120) orientation with an in-plane orientation relationship of <0001>_ZnO_//<110>_STO_ on as-received (001) STO, and polar (0001) orientation with <1100>_ZnO_//<110>_STO_ on etched (001) STO substrates, respectively. The surface energy is supposed to be dominant for *c*-axis growth on etched (001) STO. ZnO films change from polar (0001) orientation to semipolar (1012) orientation on as-received and etched (011) STO. On as-received and etched (111) STO, ZnO films show the same growth direction with polar (0001), but different in-plane orientation with 30° rotation and a large lattice mismatch induced by the extra interface chemical energy of etched (111) STO with more dangling bonds. The change of epitaxial relationship for ZnO films on as-received and etched STO substrates is accompanied with the increase of lattice mismatch, decrease of bond density, and increase of substrate surface roughness. This investigation presents a very simple way to control epitaxial relationship of ZnO films with STO substrates, which is of technological interest in optoelectronic and electronic devices.

## Competing interests

The authors declare that they have no competing interests.

## Authors’ contributions

CJ carried out the experimental analysis and drafted the manuscript. YC carried out the experimental design. XL carried out the growth and optimization of indium nitride films. SY participated in the experimental measurement. WZ participated in its design and coordination. ZW participated in the experimental design. All authors read and approved the final manuscript.
